# Cross-Domain Sentiment Analysis Based on Feature Projection and Multi-Source Attention in IoT

**DOI:** 10.3390/s23167282

**Published:** 2023-08-20

**Authors:** Yeqiu Kong, Zhongwei Xu, Meng Mei

**Affiliations:** School of Electronic and Information Engineering, Tongji University, Shanghai 201804, China; 2130694@tongji.edu.cn (Y.K.); mei_meng@163.com (M.M.)

**Keywords:** social sensor, cross-domain sentiment analysis, multi-source selection, orthogonal projection, attention mechanism

## Abstract

Social media is a real-time social sensor to sense and collect diverse information, which can be combined with sentiment analysis to help IoT sensors provide user-demanded favorable data in smart systems. In the case of insufficient data labels, cross-domain sentiment analysis aims to transfer knowledge from the source domain with rich labels to the target domain that lacks labels. Most domain adaptation sentiment analysis methods achieve transfer learning by reducing the domain differences between the source and target domains, but little attention is paid to the negative transfer problem caused by invalid source domains. To address these problems, this paper proposes a cross-domain sentiment analysis method based on feature projection and multi-source attention (FPMA), which not only alleviates the effect of negative transfer through a multi-source selection strategy but also improves the classification performance in terms of feature representation. Specifically, two feature extractors and a domain discriminator are employed to extract shared and private features through adversarial training. The extracted features are optimized by orthogonal projection to help train the classification in multi-source domains. Finally, each text in the target domain is fed into the trained module. The sentiment tendency is predicted in the weighted form of the attention mechanism based on the classification results from the multi-source domains. The experimental results on two commonly used datasets showed that FPMA outperformed baseline models.

## 1. Introduction

The Internet of Things (IoT) has become a major focus in the IT industry. IoT connects physical objects to the online world, making them virtual intelligent objects based on sensors. To simulate real-world intelligence, millions of physical objects are interconnected through sensor devices. Today, IoT is using big data sentiment analysis to reshape, analyze, and improve integrated information processing systems based on sensors [1]. Sentiment analysis empowers IoT devices to gather more useful data from massive datasets to better understand needs and optimize services, which is an important pillar for positioning and improving IoT technology. As an exemplification, social media are real-world sensors that can be employed to evaluate the pulse of societies by collecting real-time data and information from online human interactions [2,3]. The social sensor integrated with application programming interfaces for sensing news (i.e., the news sensor) combined sentiment analysis to extract sentiment information from global news and generate an interactive global threat map using geographic data [4]. This system may provide crucial decision support and intelligent early warning, allowing decision-makers to monitor the situation to identify potential hazards and improve area security by monitoring environmental conditions in real time through sensors connected to the IoT [5]. Furthermore, sentiment analysis can be combined with dynamic online user recruitment [6] to understand their sentiment tendencies and engagement levels. This information can be utilized to make decisions regarding user recruitment strategies, selecting users who exhibit a positive willingness to cooperate and higher levels of contribution.

Data from social sensors have different types of themes, and they can be considered as different domains. Currently, the approach used for sentiment analysis in a single domain is usually supervised learning of annotated samples from that domain, but this process is labor-intensive and difficult to adapt to new domains. As shown in Table 1, consider two domains: Book and Electronics. It is clear that they share common features such as “high cost–performance ratio”, while also having their own specific functional descriptions. Therefore, different domains often exhibit both shared and private features. Accordingly, the aim of cross-domain analysis is to utilize a generalized method that mines knowledge shared across domains with rich sentiment labels. This knowledge can then be used for sentiment classification in domains with few or no sentiment labels.

The fast development of IoT has significantly contributed to the promotion of sentiment analysis due to the integration of big data, cloud computing, and 5G [7]. Cross-domain sentiment analysis has attracted the attention of many scholars. For unsupervised cross-domain sentiment analysis, one solution is to continuously reduce the domain differences between the target and source via domain adaptation. Another solution is to assign weights to pre-trained source domain classifiers based on the relationship between the target and source. From the first perspective, Remus et al. [8] proposed to select samples from the source domain that were most similar to the target domain, which employed bag-of-word models for vectorization and measured similarity through the Jaccard Similarity (JS) Distance. Further introducing neural-based models, Liu et al. [9] argued that adversarial training can extract purer shared features for multi-domain text classification, which could enhance the shared feature space that only contains common and task-invariant information, without mixing unnecessary task-specific features or feature redundancy. Chen et al. [10] introduced a polynomial adversarial network that learned invariant features by reducing the differences between each domain feature distribution, which was the same as the model proposed by Liu et al. [9] when using the negative log-likelihood (NLL) loss. Moreover, Dai et al. [11] determined the source domain closest to the target domain by minimizing the A-distance between domains.

From the second perspective, Chattopadhyay et al. [12] assigned a weight to each source domain based on the conditional probability differences between the distribution of the source and target domains, but it is computationally complex. Dai et al. [13] provided a weighted strategy implemented by the discriminator through adversarial training, and the discriminator could measure the probability estimates that target instances belonged to each source domain. Fu et al. [14] calculated weights using the bidirectional Kullback–Leibler (KL) scatter between the target and source domains. Although all of the abovementioned approaches perform well, unsupervised cross-domain sentiment analysis still encounters significant challenges in terms of source domain selection and the balance between domain adaptation and private features. Previous studies [9,10,12,13,15] rarely considered the impact of source domain effectiveness on the target domain prediction, which might lower the classification accuracy.

In this paper, we not only attempt to discard invalid source domains but also continuously optimize the feature representation to extract effective features. We propose a cross-domain sentiment analysis method based on feature projection and multi-source attention. In this method, we extract shared and private features using two feature extractors and a domain discriminator. We also use orthogonal projection to purify the private features and fuse the shared features. Next, we assign weights to the source domains based on the similarity of the private features. Finally, we predict the sentiment tendency of the target domain with an attention mechanism. Furthermore, in the case of too many source domains, we exploit the probability estimates from the domain discriminator to select the source domains that more closely match the target domain. The main contributions of this paper are as follows:

- We propose a method for cross-domain sentiment analysis based on feature projection and multi-source attention (FPMA). The model optimizes the representation of the private and shared features through orthogonal projection, which enables the sentiment to be predicted based on the attention mechanism.
- We suggest a multi-source selection strategy based on the domain discriminator’s selection of the source domains that more closely resemble the text features of the target domain, effectively alleviating the negative transfer problem caused by source domains of low relevance.
- The experimental results of FPMA for both English and Chinese datasets show that the model outperforms the baseline models. We also validated the effectiveness of FPMA through ablation experiments.

Section 2 explains domain adaptation, attention mechanisms, and adversarial training in sentiment analysis. Section 3 presents the proposed method, including feature processing, multi-source classification training, multi-source selection strategy, and attention-weighted prediction. Section 4 introduces the experimental setup, and Section 5 discusses the experimental results and their analysis. Section 6 concludes the paper and describes prospects for future work.

## 2. Related Work

### 2.1. Domain Adaptation

Domain adaptation is an important part of transfer learning [16], which aims to map data from different source and target domains into a common feature space so that they are as similar as possible. Dredze et al. [17] suggested that finding a suitable domain adaptation approach would be challenging if the labeling criteria differed between domains, which is critical for domain adaptation. The source domain might be single or multiple. In single-source domain adaptation, particular emphasis is placed on overcoming distribution mismatch and domain shift difficulties. Ghifary et al. [18] utilized the maximum mean discrepancy (MMD) metric as a regularization between different domains to alleviate the distribution mismatch. With the application of deep networks, Rozantsev et al. [19] argued that the weights of the corresponding layers between the source and target domains should not be shared but associated with weight regularizers, which could automatically determine whether weights are shared or not. Xue et al. [20] introduced deep mutual learning by utilizing two groups of label probers with the same structure as sentiment classifiers, enabling the interaction of sentiment information between different groups.

In multi-source domain adaptation, more consideration should be given to how multiple source domains can be combined. Guo et al. [21] combined results from multi-source domains using a point-to-set distance and introduced meta-training to learn it. To increase effective knowledge sharing between source domains, Zhao et al. [22] utilized soft parameter sharing to capture sentiment representations across domains and obtained shared representations for the target by fine-tuning. Dai et al. [13] directly obtained the classification results of the target instances through the source domain classifier, and the weight assigned to each source domain was acquired by the domain discriminator. On this basis, Li et al. [23] used weighted private features from each source domain to strengthen the learning of private features in the target domain. Furthermore, Zhang et al. [24] found that the more similar domain features are, the more relevant instances are. Thus, feature similarity can more accurately reflect relationship information than the domain discriminator [13]. In our model, the classification labels of the target instances are still directly obtained through the source domain classifier, but the weights assigned to source domains are set by the similarity between the target instances and source domains.

### 2.2. Attention Mechanism

Attention mechanisms were first developed in the field of computer vision and were introduced into the field of natural language processing through machine translation [25] tasks. The wide range of applications of attention mechanisms in sentiment analysis tasks can effectively improve the classification efficiency of the model [26]. For example, Ji et al. [27] designed a bifurcated long short-term memory (LSTM) network using attention-based LSTM, which can extract topic and sentiment features from the source domains. Gan et al. [28] proposed a convolutional neural network (CNN)-BiLSTM model with an attention mechanism that included global and local attention, which enhanced feature differentiation. In addition, Basiri et al. [29] proposed an attention-based bidirectional CNN-recurrent neural network (RNN) depth model that applied an attention mechanism to the outputs of the bidirectional layers, allowing for varying levels of emphasis on different words. Dai et al. [30] introduced a sentence-level-based attention transfer network to address the issue of insufficient utilization of the semantic information within the sentences of a document. However, these methods applied attention mechanisms for finding key words or sentences and ignored transferable features from different source domains. In contrast, the attention mechanism designed in this paper focuses on the source domains that are useful for sentiment prediction in the target domain. The attention weights are represented by feature similarity, which can determine the importance of different source domains. Zheng et al. [31,32] demonstrated the powerful performance of Bidirectional Encoder Representations from Transformers (BERT), a pre-trained model with an attention mechanism at its core, in language representation. The BERT model is employed on a Chinese dataset to generate a text vector representation of intrinsic semantic information in our work.

### 2.3. Adversarial Training

Generative adversarial networks were proposed by Goodfellow et al. [33], who used the idea of a two-person zero-sum game to achieve an optimal equilibrium in the training. The idea of adversarial training has also been introduced for multi-domain sentiment analysis tasks. Ganin et al. [34] proposed an adversarial training process using gradient reversal layers, and the model was widely used in later studies. Adversarial training is employed to train a feature extractor that maps both the source and target domains into a shared feature space in supervised learning, allowing the classifier learned on the source data to be transferred to the target domain. For example, Ganin et al. [35] and Zhao et al. [36] both used adversarial neural networks to extract domain-invariant features. Adversarial training can also be applied to unlabeled data. Adversarial training between the classifier and the feature extractor can enhance the feature extraction capabilities for previously unseen features and strengthen the robustness of the classifier. Wu et al. [37] proposed a dual adversarial cooperative learning method that extracted domain-invariant features and ensured alignment between labeled and unlabeled data in each domain. Wu et al. [38] adopted standard adversarial training to learn domain-invariant features, and virtual adversarial training with entropy minimization to optimize the prediction inconsistency for unlabeled data. However, using adversarial training on unlabeled data may not fully capture the true features, due to the absence of label information, making the model’s performance susceptible to variations. Since the shared features obtained by adversarial training are purer [9], we obtain the shared features using adversarial training as well. Unlike previous studies, the shared features are not used directly for the classification task, and the shared and private features are further optimized via orthogonal projection to make the processed feature representation more beneficial for text classification.

## 3. Proposed Method

### 3.1. Task Description

The dataset is divided into different domains based on the topic of the texts. Suppose there are labeled text data from K source domains ${\left\{S_{i}\right\}}_{i=1}^{K}$, where $S_{i}={\left\{\left(x_{j}^{S_{i}},y_{j}^{S_{i}}\right)\right\}}_{j=1}^{\left|S_{i}\right|}$. The unlabeled target domain is denoted by $T={\left\{x_{j}^{T}\right\}}_{j=1}^{\left|T\right|}$, and the text data with domain labels are represented as ${\left\{\left(x_{j},d_{j}\right)\right\}}_{j=1}^{N}$; N denotes the total number of samples in all the source and target domains, and $N_{s}$ denotes the total number of samples in all the source domains. Table 2 provides the specific definitions of the symbols used in the above expressions. In this paper, the text sentiment is classified into positive and negative; thus, the sentiment label y can be defined as a one-hot vector. The domain label d is also represented as a one-hot vector, with a dimension equal to the total number of domains in the dataset, which is K+1. The text x described above is vectorized, and our goal is to train a text sentiment classification model for the target domain using samples from the source and target domains.

### 3.2. Framework Overview

**Figure 1:**
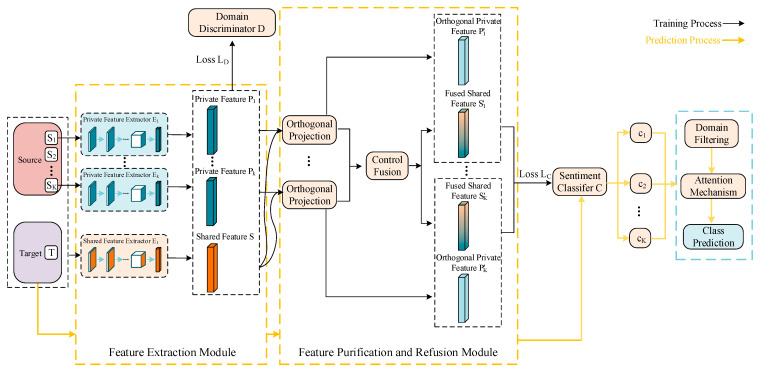
Diagram of the model structure.

We propose a method for cross-domain sentiment analysis based on feature projection and multi-source attention. The overall structure is shown in Figure 1. In the training phase, we employ two types of feature extractors and a domain discriminator to extract shared features across all the domains and private features in each source domain. Next, we purify the private features via orthogonal projection and process the shared features via controlled fusion, training the sentiment classifier for the source domains. During the prediction phase, we use the trained feature extractors to extract features for the target domain text, and directly obtain the classification labels of the target instances through the source domain classifier. The attention weights assigned to the source domain classifiers are based on feature similarity. Moreover, in the case of too many source domains, we implement domain filtering, based on a multi-source selection strategy to alleviate the negative transfer problem. In the subsequent sections, we detail feature processing, multi-source classification training, multi-source selection strategy, and attention-weighted prediction.

**Figure 2:**
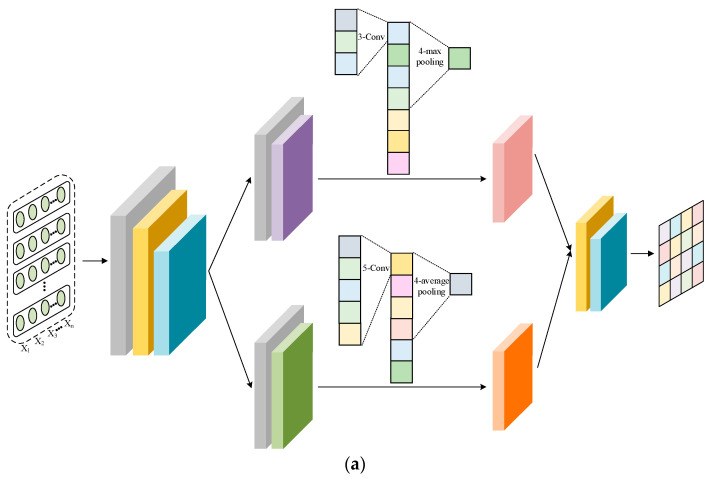

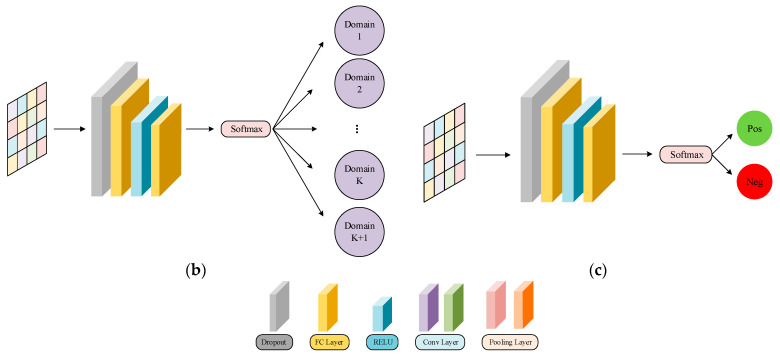
Network structure: (**a**) feature extractor, (**b**) domain discriminator, and (**c**) sentiment classifier.

The network structures of the feature extractors, domain discriminator, and sentiment classifier are shown in Figure 2. The Amazon review dataset used in the experiment had lost all of its word order information, which prevented the use of strong feature extractors such as RNN. For a fair comparison, both feature extractors consist of two fully connected (FC) layers and two parallel convolution (Conv) layers. One channel extracts local features using a Conv layer with a kernel of 3 and a max pooling layer with a kernel of 4. Another enhances global representation capability through a Conv layer with a kernel of 5 and an average pooling layer with a kernel of 4. Inconsistent kernel sizes used for convolution and pooling allow for interactive learning between features. The domain discriminator and sentiment classifier are composed of two FC layers. For domain classification, the target domain is represented as Domain K+1 in Figure 2. The dropout technique is used in all the modules to avoid overfitting.

### 3.3. Feature Processing

The feature processing step is divided into two main processes: (1) feature extraction and (2) feature purification and refusion. The former extracts the important features, while the latter further optimizes the extracted features to maximize their effectiveness for text classification.

#### 3.3.1. Feature Extraction

The feature extraction process has a domain discriminator, a shared feature extractor, and K private feature extractors. The domain discriminator expects to make correct domain judgments of the extracted features, while the shared feature extractor wants the shared features to be indistinguishable from the domain discriminator. As a result, the shared feature extractor and domain discriminator form an adversarial process. Let D denote the domain discriminator, $E_{s}$ denote the shared feature extractor, and ${\left\{E_{pi}\right\}}_{i=1}^{K}$ denote the private feature extractors for K source domains. Then, the following equations can be obtained:

(1) $$L_{adv}=-\frac{1}{N}\sum_{j=1}^{N}d_{j}^{⊤}\ln D\left(E_{s}\left(x_{j}\right)\right)$$

(2) $$L_{D\_P}=-\frac{1}{N_{s}}\sum_{i=1}^{K}\sum_{j=1}^{\left|S_{i}\right|}d_{j}^{⊤}\ln D\left(E_{pi}\left(x_{j}^{S_{i}}\right)\right)$$

(3) $$L_{D}=L_{adv}+L_{D\_P}$$

After adversarial training, the shared feature extractor extracts purer shared features, and the domain discriminator learns which domain the input features come from, enabling an improved separation between private features and shared features. It should be stressed that the discriminator plays a crucial role in the multi-source selection strategy to filter out ineffective source domains.

#### 3.3.2. Feature Purification and Refusion

Although the shared features obtained by the adversarial training are purer, certain shared features may still be intermingled with the private features of each source domain, degrading the sentiment classification. Inspired by Qin et al. [39], this work attempts to purify private features via orthogonal projection.

**Figure 3:**
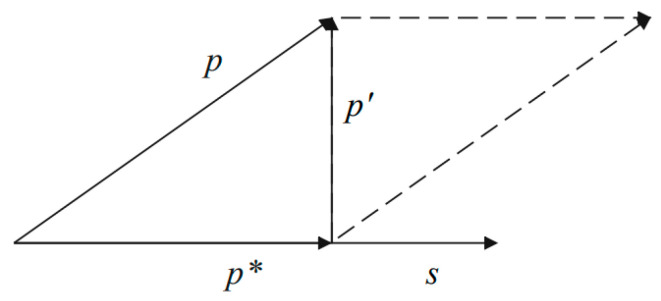
Orthogonal projection.

The process of two-dimensional orthogonal projection is shown in Figure 3. For an input text vector x, $s=E_{s}\left(x\right)$, which is a shared feature, and $p=E_{p}\left(x\right)$, which is a private feature. Firstly, p is projected onto s to obtain p*, and then p is projected onto the orthogonal direction of s to obtain p', which is the purified private feature. The process is as follows:

(4) p*=Proj(p,s)

(5) p'=Proj(p,(p−p*))

where the general equation for orthogonal projection is

(6) $$\mathrm{Proj}\left(a,b\right)=\frac{a\cdot b}{\left\|a\right\|}\frac{b}{\left\|b\right\|}$$

**Figure 4:**
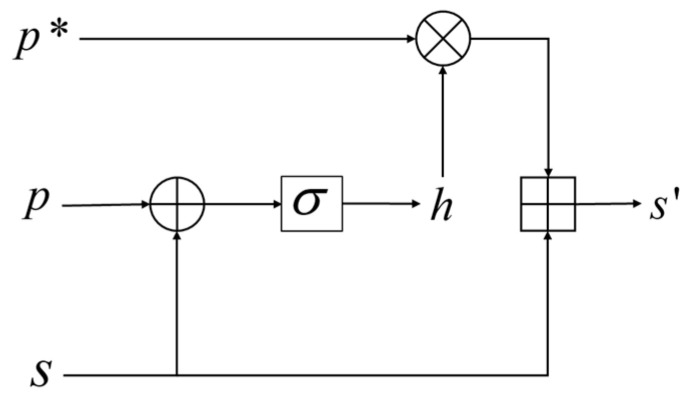
Refusion process.

In addition, the shared features mixed in with the private features may be redundant or may not be extracted by the shared feature extractor. The orthogonal projection shown in Figure 3 also yields a feature, p*, in the same direction as the shared features; thus, it may have the same or different parts as the shared feature s. As illustrated in Figure 4, we fuse p* and s,

The formulas for the above process are:

(7) h=σ(W[p⊕s])

(8) s'=h⊙p*+s

where W represents the weight vector involved in training, σ represents the sigmoid function, ⊕ represents the concatenation of features, and ⊙ represents the corresponding element-by-element multiplication. The above process automatically controls the selection of p* based on the private and shared features, and then adds the selected portion to s to obtain the fused shared features, s'.

To simplify the depiction of the processes, we designate the fusion process of shared features s as Φ and the purification process of private features p as Ψ. The preceding steps can then be simplified as Φ(s,p) and Ψ(s,p), respectively.

### 3.4. Multi-Source Classification Training

The input to the source domain sentiment classifier is the concatenation of the purified private features and the fused shared features described above. If C denotes the source domain sentiment classifier, and ⊕ denotes the concatenation of features, then, for the inputs from source domains, the following equation can be obtained:

(9) $$L_{C}=-\frac{1}{N_{s}}\sum_{i=1}^{K}\sum_{j=1}^{\left|S_{i}\right|}y_{j}^{S_{i}⊤}\ln C\left(\Phi \left(E_{s}\left(x_{j}^{S_{i}}\right),E_{pi}\left(x_{j}^{S_{i}}\right)\right)⊕\Psi \left(E_{s}\left(x_{j}^{S_{i}}\right),E_{pi}\left(x_{j}^{S_{i}}\right)\right)\right)$$

The training process for FPMA is divided into two parts. After initializing all the network parameters, the first step is the training of the domain discriminator, which enhances its discrimination ability by minimizing adversarial losses. The second step is the training of the feature extractors and the sentiment classifier, updating the parameters through adversarial loss and sentiment classification loss. The weight coefficient λ is a trade-off between these two forms of loss, and the loss function is defined as follows:

(10) $$L_{total}=L_{C}-\lambda L_{adv}$$

According to the practice commonly adopted for generative adversarial networks, the domain discriminator is trained first, and then its parameters are fixed before training others. The specific process is shown in Algorithm 1.
**Algorithm 1.** Module training process**Input:**     samples with sentiment label y for the source domains {S};
     samples for the target domain {T};
     samples with domain label d for all the domains {A};
**Output:**
     Optimized parameter set $\theta_{opt}$;
1:Initialize parameter set θ;2:repeat3:  for t=1 to n do4:    $L_{D}=0$5:    for d∈{A} do6:      Take b samples ${\left(x_{j},d_{j}\right)}_{j=1}^{b}∼\left\{A\right\}$7:      Extract shared features $E_{s}\left(x_{j}\right)$8:      Calculate adversarial loss $L_{adv}$ using Equation (1)9:      if d∈{S} then10:        Extract private features $E_{pi}\left(x_{j}\right)$ based on the corresponding source domain11:        Calculate private loss $L_{D\_P}$ using Equation (2)12:      Calculate loss $L_{D}$ using Equation (3)13:      Update parameters of the domain discriminator by minimizing $L_{D}$14:    end for15:  end for16:  $L_{total}=0$17:  for d∈{S} do18:    Take b samples ${\left(x_{j},y_{j}\right)}_{j=1}^{b}∼\left\{S\right\}$19:    Calculate two orthogonal projections to purify private features and fuse shared features using Equations (4)–(8)20:    Calculate sentiment classification loss $L_{C}$ using Equation (9)21:  end for22:  for d∈{A} do23:    Calculate adversarial loss $L_{adv}$ using Equation (1)24:  end for25:  $L_{total}=L_{C}-\lambda L_{adv}$26:   Update parameters of the shared feature extractor, private feature extractor, and sentiment classifier by minimizing $L_{total}$27:return optimized parameter set $\theta_{opt}$


### 3.5. Multi-Source Selection Strategy

**Figure 5:**
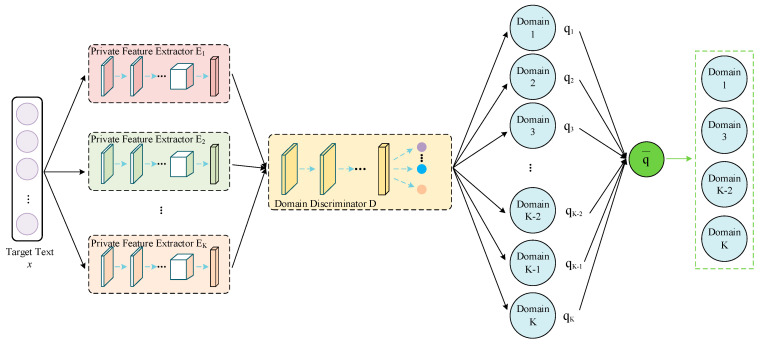
Domain filtering.

The text data in the source domain can provide rich feature information to the target domain, but certain source domain samples may make negative contributions to the domain adaptation; this is called negative transfer. In response, this paper proposes a multi-source selection strategy, as shown in Figure 5.

A text vector from the target domain is sequentially fed into the trained private feature extractors and domain discriminator, and the probability distribution estimates $\left[q_{1},q_{2},\cdots ,q_{K}\right]$ belonging to each source domain are produced as output. If the probability distribution is relatively uniform (i.e., all the source domains can provide references for the classification prediction), then no further filtering is required. If the probability distribution is not uniform enough, the text may be more closely related to particular source domains, and the source domains with higher probability values must be selected to alleviate the effect of negative transfer. In the abovementioned process, the uniformity of the probability distribution can be measured by the standard deviation, which is given by the following equations:

(11) $$s=\sqrt{\frac{\sum_{i=1}^{K}\left(q_{i}-\bar{q}\right)}{K}}$$

(12) $$\bar{q}=\frac{\sum_{i=1}^{K}q_{i}}{K}$$

The multi-source selection is performed only when the standard deviation exceeds a certain threshold, and the source domains with a probability higher than the average probability $\bar{q}$ are selected. The threshold value is adjusted according to the experimental results, and we set it to 0.043. Moreover, the object of the multi-source selection strategy is each text in the target domain. Each text selects different source domains according to its own characteristics, which makes the strategy more tailored and improves the accuracy of the classification prediction.

### 3.6. Attention-Weighted Prediction

**Figure 6:**
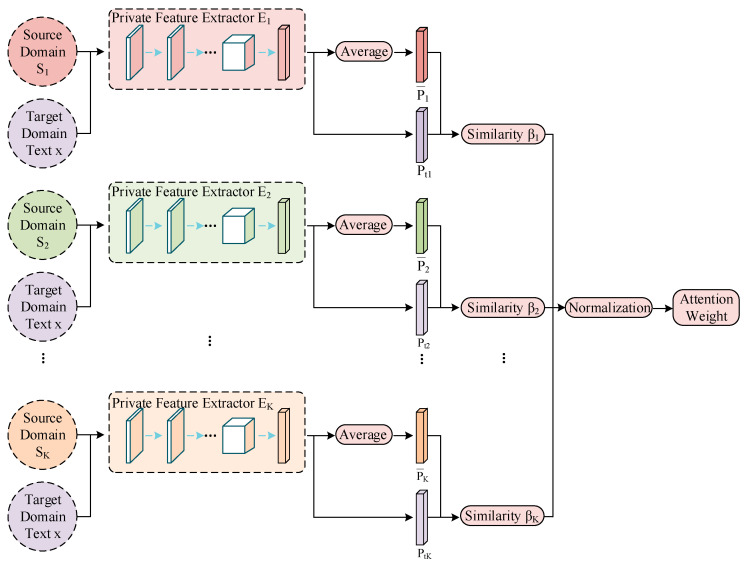
Calculation of attention weights.

The key to weighted prediction is to find the correlation between the source domains and each text in the target domain. First, all the texts in each source domain are assigned private features through the corresponding domain private feature extractor. Averaging these private features (i.e., the average private feature) captures a large amount of feature information from the domain. Thus, the average private feature can be regarded as a typical feature of each domain. Second, when a text from the target domain passes through the trained private feature extractors, K different private features can also be obtained. The similarity between these two types of private features can be calculated separately to measure the correlation between the text and different source domains, obtaining the corresponding attention weight coefficients for different source domains. The specific process is shown in Figure 6.

The attention mechanism is divided into two main steps: (1) calculating the weight coefficients based on the query and key vectors, and (2) weighting the value vectors according to the weight coefficients and summing them. The first step can be subdivided into two stages: (1) calculating the similarity or correlation between the query and key vectors, and (2) normalizing the original scores from the first stage. Therefore, let K private features of the target domain $\left[p_{t1},p_{t2},\cdots ,p_{tK}\right]$ be the query vector and K average private features of the source domains $\left[\bar{p_{1}},\bar{p_{2}},\cdots ,\bar{p_{K}}\right]$ be the key vector. The trained sentiment classifier is directly applied to a text in the target domain to obtain the classification results $\left[c_{1},c_{2},\cdots ,c_{K}\right]$, which are used as the value vector. This work uses cosine similarity to calculate the relevance of the private features in the target domain and the average private features in the source domains. The formulas are expressed as

(13) $$sim\left(p_{ti},\bar{p_{i}}\right)=\frac{p_{ti}\cdot \bar{p_{i}}}{\left\|p_{ti}\right\|\left\|\bar{p_{i}}\right\|}$$

(14) $$\alpha_{i}=\frac{\exp\left(sim\left(p_{ti},\bar{p_{i}}\right)\right)}{\sum_{j}\exp\left(sim\left(p_{tj},\bar{p_{j}}\right)\right)}$$

(15) $$c_{t}=\sum_{i}\alpha_{i}c_{i}$$

where $c_{t}$ is the sentiment classification result for the target domain text. Using private features rather than shared features to calculate similarity can better apply domain features to the target domain. The attention mechanism cleverly accounts for the relative importance of different domain features, assigning larger weights to the sources that generate important features and smaller weights to the sources that produce unimportant or irrelevant features. As a result, the full utilization of different features from different perspectives significantly benefits the final classification results.

## 4. Experiments

### 4.1. Datasets Used in the Experiment

The Amazon review dataset [40] is one of the most widely used datasets in text classification. Each text in the dataset is originally represented as a 5000-dimensional vector using bag-of-word unigram and bigram features. The dataset contains four domains. Each domain has 1000 positive samples, 1000 negative samples, and a group of unlabeled samples, as shown in Table 3. In this paper, three domains are used as the source domains, and the remaining one is used as the target domain. All the samples in the source domains are taken as the training set, the labeled samples in the target domain are taken as the validation set, and the unlabeled samples in the target domain are taken as the test set.

The online_shopping_10_cats dataset is a commonly used Chinese sentiment classification dataset. It is divided into 10 categories. The number of positive and negative reviews is about 30,000 each, as shown in Table 4. Due to the small sample size of the water heater category and the extremely unbalanced distribution of positive and negative samples, training it as a single domain is challenging. Therefore, we conducted experiments only on the remaining nine domains. Eight domains are sequentially selected as the source domains, and the remaining domain is selected as the target domain. All the samples in the source domains are taken as the training set to train the model, while all the samples in the target domain are equally divided into two parts: (1) the validation set and (2) the test set. A vectorized representation of the Chinese texts was generated with BERT, whose vector dimension is 768.

### 4.2. Experiment Settings

BERT consisted of 12 layers, 768 hidden units, and 12 attention heads. All the networks used the Adam optimizer in the parameter optimization process, with an initial learning rate of 0.0001 and a batch size of 64. The weight coefficient, λ, of the loss function was fine-tuned according to the actual situation. The output size of both feature extractors was 128. The hidden layer size of both the domain discriminator and the sentiment classifier was 64. For the Amazon review dataset, λ was set to 0.22, and the dropout ratio was set to 0.4. The output size of the first FC layer in the feature extractor was 1000. The output sizes of the Conv layer and the pooling layer in channel one were 1000 and 250, respectively. Channel two was identical to channel one. For the online_shopping_10_cats dataset, λ was set to 0.40, and the dropout ratio was set to 0.2. The output size of the first FC layer of the feature extractor was 384. The output sizes of the Conv layer and the pooling layer were 384 and 96, respectively. In addition, early stopping was implemented on the validation set in the training process.

### 4.3. Baseline Models

In order to verify the effectiveness of FPMA, experiments were conducted to compare it with several baseline models. The amount of data in the dataset is relatively balanced; thus, we used classification accuracy to evaluate the performance of the various methods. We used a multi-source selection strategy on the online_shopping_10_cats dataset but not on the Amazon review dataset for its limited number of domains.

The baseline models for the Amazon review dataset included:

- mSDA [41]: marginalizes the noise through a domain adaptation edge denoising self-encoder without using any optimization algorithm to learn the parameters in the model.
- DANN [35]: extracts domain-invariant features via domain adversarial neural networks.
- MDAN(Hard-Max), MDAN(Soft-Max) [36]: two adversarial neural network models; the former optimizes the domain adaptation generalization boundary, and the latter is a smooth approximation of the former.
- MAN [10]: learns invariant features by reducing the difference between the distribution of features in each domain.
- MDAJL [22]: employs a framework with joint learning that uses soft parameter sharing for cross-task information transfer.
- HM-LTS [42]: combines a lexicon-based unsupervised method, a support vector machine-based supervised method, and topic modeling.
- SDA [11]: uses a shared–private structure to transfer knowledge from multi-source domains through two domain adaptation mechanisms.

The baseline models for the online_shopping_10_cats dataset included:

- BTDNNs [43]: transfers the samples in the source and target domains to each other, constraining the distribution consistency between the transferred and desired domains via linear data reconstruction.
- MDAN [36]: uses domain adversarial neural networks to optimize the domain adaptation generalization boundary.
- WS-UDA [13]: an unsupervised framework based on a weighted scheme; the weight assigned to each source is acquired from the domain discriminator via adversarial training.
- 2ST-UDA [13]: further utilizes the pseudo labels of the target domain to train a target private extractor on the basis of WS-UDA.
- AdEA [23]: utilizes a weighted learning module to strengthen the relationship between domain features.

## 5. Experimental Results and Analysis

### 5.1. Main Experimental Results

Table 5 presents a comprehensive analysis of the classification accuracies and average accuracies obtained by the baseline models and FPMA on the Amazon review dataset, with the best outcomes highlighted in bold. FPMA outperformed all the baseline models in terms of average accuracy, with a 0.97% improvement over SDA (which has the highest average accuracy among the baseline models) and a 1.33% improvement over the second-highest model. Moreover, FPMA exhibited exceptional performance in all four domains by achieving the highest accuracies. Unlike most baseline models, it can handle multiple target domains simultaneously. This characteristic enhances the practical applicability and versatility of FPMA in real-world scenarios with diverse domains and datasets.

Table 6 presents the classification accuracy and average accuracies of the baseline models and FPMA for the online_shopping_10_cats dataset, with the best outcomes highlighted in bold. FPMA improved the average accuracy by 1.6% compared with AdEA, which had the highest average accuracy in the baseline models. Additionally, when compared with the previous top-performing model in each target domain, FPMA exhibited improvements in classification accuracies for seven out of the ten target domains, with enhancements of 1.6%, 5.0%, 0.2%, 0.4%, 2.6%, 0.3%, and 7.3%, respectively. However, it is worth mentioning that the accuracy for the Book category was relatively lower. This observation can be attributed to the nature of the reviews in the Book domain, which often contain a significant number of book titles. While BERT excels at processing semantic information, it may not be well-suited for scenarios with strong noun characteristics. As a result, the text vectors derived from BERT may deviate from the original semantic information, thereby impacting the accuracy of feature representation in this particular domain. Compared with these baseline models, our multi-source selection strategy employs a more targeted approach to identify source domains that closely align with the characteristics of the target domain. This strategic selection allows the target domain’s text to concentrate attention on highly transferable source domains during the classification process, demonstrating improved performance and enhanced feature representation.

### 5.2. Ablation Experiments

**Figure 7:**
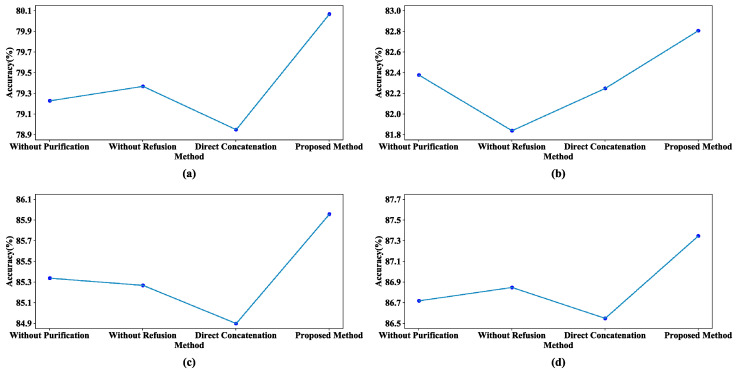
Comparison results of different methods on the Amazon review dataset: (**a**) Book, (**b**) DVD, (**c**) Electronics, and (**d**) Kitchen.

To further validate the effectiveness of FPMA, we conducted ablation experiments on two datasets. Figure 7 shows the results of four different experiments on the Amazon review dataset, verifying the effectiveness of feature optimization processing. In the figure, “Without Purification” indicates that no purification of private features was performed, “Without Refusion” indicates that the shared features were not fused, and “Direct Concatenation” indicates that the private and shared features were simply concatenated together without processing. Figure 7 illustrates that the accuracies for all four domains experienced varying degrees of decline after one or more of the feature processing mechanisms was removed. Moreover, these models consistently failed to reach the performance exhibited by FPMA in all the domains. This observation strongly suggests that the purification of private features and the refusion of shared features can filter out the negative effects of inefficient features, improving the classification accuracy of the model.

**Figure 8:**
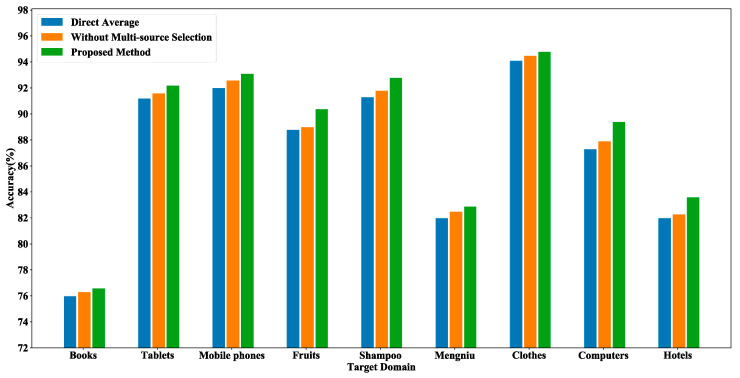
Comparison results of different methods on the online_shopping_10_cats dataset.

Figure 8 shows the results of three different experiments on the online_shopping_10_cats dataset, aiming to validate the effectiveness of the multi-source selection strategy. In the figure, “Direct Average” represents the direct averaging of the prediction results obtained from all the source domains, and “Without Multi-source Selection” indicates that the experiment used the classification results of all the source domains to make predictions on the target domain text. Figure 8 demonstrates that the accuracy of using all the source domains weighted was slightly higher than the prediction result of direct averaging. After utilizing the multi-source selection strategy, we only implemented the attention mechanism for the filtered source domains, and the accuracy of the prediction results reached the highest out of all nine domains. It is obvious that the multi-source selection strategy can find the source domains that are more favorable to the target domain text classification. It effectively alleviates the effect of negative transfer caused by less relevant source domains, and each text in the target domain selects different source domains based on similarity, which can be more targeted in the prediction process.

**Figure 9:**
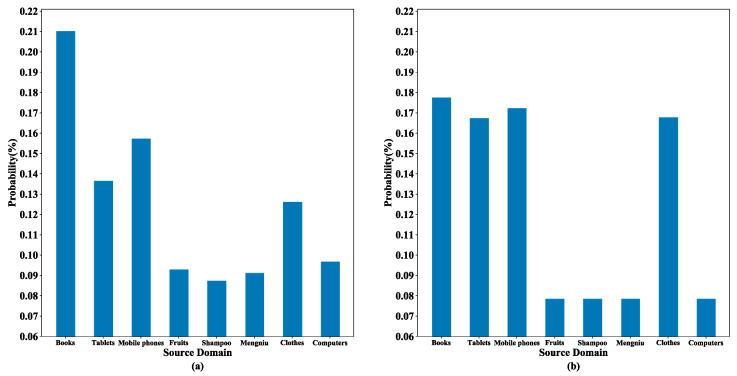
Distribution comparison chart: (**a**) probability estimation of the domain discriminator, and (**b**) weights of the source domains.

Figure 9 shows the probability distribution for an experiment in which the computer domain is used as the target domain and the other eight domains as the source domains. The left panel shows the probability estimates of the domain discriminator for a text in the computer domain, and the right panel shows the source domain weight distribution obtained according to the multi-source selection strategy and private feature similarity. In the left panel, it can be observed that four of the eight source domains had significantly higher probability estimates, suggesting that these four domains had a strong match with this text in the target domain. Thus, these four domains were reserved using the multi-source selection strategy. In the right panel, the four source domains that had been reserved received greater weights after combining the private feature similarity, while the other domains obtained very small weights through the normalization process. Importantly, this process did not over-strengthen the source domains with previously higher probability estimates (i.e., the corresponding book category in the figure). It effectively addressed the challenge of balancing domain adaptation and private features, enabling the consideration of both aspects in determining the optimal weight allocation method for the target domain text.

## 6. Conclusions

This paper proposes a cross-domain sentiment analysis method based on feature projection and multi-source attention, aiming to empower the IoT industry to leverage big data sentiment analysis with improved classification accuracy. The model extracted shared and private features using a domain discriminator. For improved classification, it also employed feature projection to further fuse and purify the shared and private features. To alleviate the effect of negative transfer caused by invalid domains, this study also offered a multi-source selection strategy. This strategy selected the source domains with greater correlation to each text in the target domain for classification prediction. FPMA outperformed previous baseline models in terms of average accuracy for both the Amazon review and the online_shopping_10_cats datasets. Moreover, the effectiveness of the feature processing and the multi-source selection strategy was verified through ablation experiments. This framework can benefit the IoT community through more effective sentiment analysis using big data. Performing sentiment analysis on data from social sensors can better drive IoT sensors to obtain valuable data that meet user demands, enabling the realization of various intelligent systems such as smart homes.

In future work, we will further explore applications for datasets with richer sentiment labels. Furthermore, we intend to enable FPMA to perform multi-modal analysis, which considers the fusion of different types of data from multiple sensors. In terms of method application transfer, it is also challenging to collect sufficient data for a newly launched system to train an unsupervised log anomaly detection model. Similar to cross-domain sentiment analysis, a new system often requires log data from other systems for log anomaly prediction. We will continuously improve FPMA and apply it to log anomaly detection.

## Figures and Tables

**Table 1:** Reviews from Book and Electronics domains.

Domain	Review	Sentiment
Book	The book is rich in content and has a high cost–performance ratio.	positive
Electronics	The mobile phone offers a high cost–performance ratio, comprehensive functions, and a long standby time.	positive

**Table 2:** Symbols and definitions.

Symbols	Definitions
$x_{j}^{S_{i}}$	j-th text in the i-th source domain
$y_{j}^{S_{i}}$	sentiment label of the j-th text in the i-th source domain
$\left|S_{i}\right|$	number of samples in the i-th source domain
$x_{j}^{T}$	j-th text in the target domain
|T|	number of samples in the target domain
$d_{j}$	domain label corresponding to the j-th text

**Table 3:** Amazon review dataset.

Categories	Positive Samples	Negative Samples	Unlabeled Data
Book	1000	1000	4465
DVD	1000	1000	3586
Electronics	1000	1000	5681
Kitchen	1000	1000	5945

**Table 4:** Online_shopping_10_cats dataset [23].

Categories	Positive Samples	Negative Samples
Books	2100	1751
Tablets	5000	5000
Mobile phones	1165	1158
Fruit	5000	5000
Shampoo	5000	5000
Water heaters	475	100
Mengniu	992	1041
Clothes	5000	5000
Computers	1996	1996
Hotels	5000	5000

**Table 5:** Classification accuracy results for the Amazon review dataset.

Target Domain	Book	DVD	Electronics	Kitchen	Average
mSDA [41]	76.98	78.61	81.98	84.26	80.46
DANN [35]	77.89	78.86	84.91	86.39	82.01
MDAN (Hard-Max) [36]	78.45	77.97	84.83	85.80	81.76
MDAN (Soft-Max) [36]	78.63	80.65	85.34	86.26	82.72
MAN-L2 [10]	78.45	81.57	83.37	85.57	82.24
MAN-NLL [10]	77.78	82.74	83.75	86.41	82.67
MDAJL [22]	78.80	80.20	81.20	54.30	73.60
HM-LTS [42]	74.00	76.00	79.00	80.00	77.25
SDA [11]	78.68	81.23	85.06	87.33	83.08
FPMA	**80.07**	**82.81**	**85.96**	**87.35**	**84.05**

Bold fonts indicate the best results.

**Table 6:** Classification accuracy results for the online_shopping_10_cats dataset.

Target Domain	Books	Tablets	Mobile Phones	Fruit	Shampoo	Mengniu	Clothes	Computers	Hotels	Avg.
BTDNNs [43]	78.2	85.6	82.1	87.5	88.1	73.9	91.4	80.3	78.7	82.9
MDAN [36]	78.4	86.8	81.4	87.3	87.9	73.8	91.5	79.4	80.7	83
WS-UDA [13]	77.7	90	87	89.9	91.7	76.6	94.5	81.1	82.9	85.7
2ST-UDA [13]	**82.2**	89.9	82.7	89.5	91.4	80.3	94.1	76.9	82.4	85.5
AdEA [23]	82	90.6	88.1	90.2	92.4	76.6	94.4	82.1	**84.3**	86.8
FPMA	76.6	**92.2**	**93.1**	**90.4**	**92.8**	**82.9**	**94.8**	**89.4**	83.6	**88.4**

Bold fonts indicate the best results.

## Data Availability

The datasets are publicly available. They can be found in the URLs: http://www.cs.jhu.edu/~mdredze/datasets/sentiment and https://github.com/SophonPlus/ChineseNlpCorpus/tree/master/datasets/online_shopping_10_cats (accessed on 15 August 2023).
